# A Phenomenological Investigation of the Interplay Among Professional Worth Appraisal, Self-Esteem and Self-Perception in Nurses: The Revelation of an Internal and External Criteria System

**DOI:** 10.3389/fpsyg.2018.01805

**Published:** 2018-10-01

**Authors:** Maria Karanikola, Karolina Doulougeri, Anna Koutrouba, Margarita Giannakopoulou, Elizabeth D. E. Papathanassoglou

**Affiliations:** ^1^Department of Nursing, School of Health Sciences, Cyprus University of Technology, Limassol, Cyprus; ^2^Department of Industrial Engineering and Innovation Sciences, Eindhoven University of Technology, Eindhoven, Netherlands; ^3^Department of Nursing, National and Kapodistrian University of Athens, Athens, Greece; ^4^Faculty of Nursing, University of Alberta, Edmonton, AB, Canada

**Keywords:** self-concept, personal effectiveness, professional value, self-value, burnout, qualitative study, internal criteria, external criteria

## Abstract

Nurses’ professional self-concept is strongly associated with professional worth appraisal, which encompasses their feelings and perceptions regarding their task efficacy and value of input to clinical outcomes. Professional self-concept and professional worth appraisal are incorporated in one’s overall professional role perception. Data show that the way nurses think and feel about themselves personally and professionally, is associated with their well-being, the quality of provided patient care, their job satisfaction and retention. Although researchers indicate that professional self-concept is a different entity from personal self-concept, however, a clear differentiation and possible interaction between these constructs has not been yet adequately described in nursing literature. Personal self-concept mirrors the way people interpret them-selves, incorporating their self-awareness and personal effectiveness. Following purposeful sampling and informed consent, a phenomenological approach based on Munhall’s methodology was employed to explore the living experience of professional role perception in 16 critical and emergency nurses, with special focus on their perceptions and feelings about personal and professional-role worth appraisal. Data and theoretical saturation criteria were implemented, along with all nine Munhall’s criteria for the rigor and trustworthiness of phenomenological studies. The participants’ narratives suggested a possible interaction between professional attitude and personality traits, illuminating as the core theme an interplay among self-perception, personal and professional worth appraisal process. Additionally, the present study emphasized the way self-evaluation criteria system may be associated with the personal and professional self-concept in nurses. In particular, it was highlighted that the way nurses think and feel about themselves is associated with the way they experience their professional role and vice versa, and that professional role-based self-concept and professional worth perception can be linked with their well-being. Furthermore, positive feelings about the self and personal competencies seemed to enhance the perception of effectiveness in clinical settings and adequacy of professional skills, resulting in empowered professional identity and vice versa. Overall, the present findings are discussed in relation to nurses’ experience of work-related stressors and relevant interventions. Further exploration of the effectiveness of interventions for facilitating adaptive personal and professional self-appraisal are suggested.

## Introduction

The way nurses think and feel about themselves has important implications at a personal and a professional level, i.e., well-being status, quality of provided patient care, job satisfaction and job retention (Bornholt and Piccolo, 2005; Cao et al., 2015, 2016; Caza and Creary, 2016). These effects have been associated with patient safety issues (Aiken et al., 2001; Andrews et al., 2011). Nurses’ perception of their competencies, skills and professional worth is crucial for achieving positive performance standards (Björkström et al., 2008). Professional self-concept (or professional role-based identity) is considered as self-evaluation of one’s professional knowledge, values, motives and skills (Sedikides and Brewer, 2001; Caza and Creary, 2016). Thus, nurses’ professional self-concept is strongly associated with professional role-based worth appraisal, which encompasses their feelings and perceptions regarding their task efficacy and value of input to clinical outcomes (Angel et al., 2012). Professional self-concept and professional role-based worth appraisal are incorporated in one’s overall professional role perception (Caza and Creary, 2016).

Personal self-concept (or self-perception or personal role-based identity) entails one’s personal assessment and awareness with regard to social, cognitive and physical attributes of her/his existence (Marsh et al., 2010). Thus, personal self-concept mirrors the way one thinks and feels about his/her self, thereby incorporating one’s self-awareness, personal esteem and self-confidence (Marsh et al., 2010). Personal esteem or personal role-based worth appraisal reveals the way one assesses his/her own value. Thus, self-concept may be viewed as an internal, personality standard that partially forms and regulates the behavior and performance of an individual, including professional activities (Zlatkovic et al., 2012).

Although researchers indicate that personal self-concept is a different entity from professional self-concept, however, a clear differentiation and possible interaction between these constructs have not been yet adequately described in nursing literature (Johnson et al., 2012). At the same time, there is limited data on the association between personal self-concept and nurses’ professional attitudes (Parandavar et al., 2015); however, it could be expected the opposite since personal self-concept is associated with a number of factors related to productivity and effective clinical practice, such as the development of goals, expectations and behaviors (Sommer and Baumeister, 2002). Moreover, although it is well stated that personal self-concept is a criterion of personal worth appraisal, however, the association between personal self-concept and self-assessment of professional worth or professional self-concept has not been explored adequately (Sim et al., 2014; Chang and Yeh, 2016). Zlatkovic et al. (2012) reported that self-evaluated efficacy in one’s professional role is related with his/her self-perceived total self-efficacy. Total self-efficacy is a crucial dimension of personal self-concept which reflects one’s subjective feelings and assessment of competency for everyday actions and effective performance. However, relevant data have not been explored adequately in nursing literature.

Several determinants may influence nurses’ personal and professional self-concept, including work environment, personal and professional values, education and culture, as well as interaction with colleagues, other healthcare professionals and patients (Ewens, 2003; Kantek and Şimşek, 2017; Çöplü and Tekinsoy Kartın, 2018). Other data show that nurses partially form some characteristics of their professional self-concept from their public image (Emeghebo, 2012; ten Hoeve et al., 2014). Public image portrays society’s appraisal of the qualities and status of a social group (Takase et al., 2006). Until recently, our understanding of the nursing image has been mainly based on perspectives of others such as the public or media, rather than nurses themselves (Fletcher, 2007). A number of studies have examined nurses’ and nursing students’ perceptions of the public image of the nursing profession, as well as the features of nurses’ professional identity (Johnson et al., 2012). Furthermore, other studies exploring nurses’ perceptions and feelings about their public image, reveal a discrepancy between nurses’ perceptions of their professional worth and social appraisal of nursing. This incongruity seems to cause feelings of disappointment and dissatisfaction in nurses (Errasti-Ibarrondo et al., 2012). Another study revealed that, although nurses were viewed by the public as feminine and caring professionals, they were not recognized as leaders or independent healthcare professionals (Takase et al., 2006). Regarding job retention, research indicates that nurses choose to leave their profession due to adverse changes in their personal or professional esteem and self-concept (Takase et al., 2006; Cowin et al., 2008). It seems that a negative public image of the nursing profession may adversely influence nurses’ personal and professional self-concept (ten Hoeve et al., 2014).

A deeper focus on the development of professional role perception and revelation of a possible link to personal self-concept and the worth appraisal process would give a constructive understanding of the way professional attitudes are influenced (Benner et al., 2009). Thus, exploring professional role perception, along with personal concept indices in nurses is important to ensure nurses not only remain within the profession, but also continue to provide a high quality of care.

### Aim

The aim of the current study was to qualitatively explore the living experience of professional role perception, especially focusing on perceptions and feelings about personal and professional self-concept in Greek critical and emergency nurses. Although the term “lived experience” is commonly used in the qualitative research literature, we introduce and propose the term “living experience” as more accurate, since participants appear to relive their experiences while reflecting upon them through their narratives at the time of the interview. Additionally, all participants were active clinical nurses at the time of the study, reflecting on both the past and the present.

## Materials and Methods

A phenomenological approach based on Munhall (1994) was employed. Munhall’s approach has its underpinnings in van Manen (1990) phenomenology. Munhall’s methodology was selected as most appropriate to explore perceptions and feelings of Greek hospital nurses about their professional role and personal and professional role-based worth appraisal, as it is designed for the interpretation of the living experiences of human phenomena in nursing. According to Munhall (1994), phenomenology regards the study of the world as it is experienced by those who live it, thus as a research methodology is suitable for the exploration of the living experience of everyday life situations, e.g., professional life situations. With regard to the nursing profession, the focus of phenomenology is considered as the description and revelation of the caring phenomenon, as well as the living experience of those who are part of the healthcare system, including clinicians. The method of Munhal is both descriptive and interpretative (hermeneutic). Thus, the aim of phenomenological studies is to explore the subjective interpretation of a living experience and disclose individuals’ perceptions and meanings through their lifeworld narratives. Specifically, Munhall’s methodology involves the description of the way one orients oneself into a phenomenon, for example how individuals describe the different dimensions of their professional role, also focused on the interpretation the living experience with this phenomenon in its essences, for example how individuals perceive themselves within their professional role.

### Research Ethics

A written informed consent was obtained from all participants before and after the research process, including: (a) full disclosure of the research procedure, (b) confirmation of participants’ willingness to participate in the study, and (c) reassurance of their right to withdraw freely. The project was approved by the Committee of Ethics of the Department of Nursing of the National and Kapodistrian University of Athens.

### Sampling

Purposive sampling was employed (Morse, 2015). The inclusion criteria were: (a) sufficient nursing clinical practice in a critical or emergency setting (at least of 2-year duration) and (b) willingness and ability to communicate and reflect on the living experience of nursing, as drawn from the narrative content (Morse, 2015). The sample size of 16 participants was based on the criterion of theoretical and data saturation of emergent themes (Munhall, 1994; Morse, 2015). Participants were all registered bachelor nurses, employed in public, adults’ hospital nursing services. Their characteristics (demographics, employment, and educational data) are presented in **Table 1**.

**Table 1:** Demographic, educational and work-related characteristics of the participants (*n* = 16).

Variable	Categories	Percentage (frequency)	
Gender	Male	0.06% (1)	
	Female	93.7% (15)	
Education	Bachelor degree (4-year education)	37.5% (6)	
	Associate diploma (4-year education)	62.5% (10)	
	Master’s holders	43.7% (7)	
	Ph.D. candidate	0.06% (1)	
Family status	Single	62.5% (10)	
	Married	31.2% (5)	
	Divorced	0.06% (1)	
Ranking	Staff nurse	75.0% (12)	
	Senior nurse	25.0% (4)	
Work setting	Medical-surgical	25.0% (4)	
	Intensive care unit	50.0% (8)	
	Emergency room	25.0% (4)	

**Variable**	**Mean value *(SD)***	**Minimum value**	**Maximum value**

Age (years)	34.8 (2.6)	25	45
Total work experience in nursing (years)	12.1 (1.3)	2,5	20

### Data Collection

Data were collected through repeated individual phenomenological interviews. Each interview was based on a question guide encompassing open-ended questions according to the aim of the present study and relevant literature (Munhall, 1994; Takase et al., 2006; ten Hoeve et al., 2014). All interviews were carried out by the primary investigator. The focus was on the living experience of nurses’ professional role and their appraisal of personal and professional worth. On average, three interviews were conducted with each participant, ranging from 1 to 3 h in duration. During the second interview additional information and clarifications were established, while in the third interview the emerging themes were confirmed and enriched.

The following questions were posed to participants, exactly as stated below:

(1) “Please relate to me about being a nurse.”
(2) “Please describe the way you perceive your self as an individual, as well as a nurse.”
(3) “Please describe the way you appraise your personal and professional worth.”
(4) “How do you feel about your self/personal and professional worth?”

### Data Analysis

Data collection and analysis evolved through the stages of intuition, analysis, and description (van Manen, 1990). Prior to the commencement of data collection, researchers reflected on their own experiences, beliefs and meanings of nursing in order to identify and subsequently control, to a certain degree, their subjective interpretations. This technique was used to help researchers unveil all possible experiences of participants, through analysis, as well as to assure the rigor of the analysis. Analysis was performed by three investigators (MK, AK, EP), who worked independently, and involved the identification of the nature of personal and professional worth appraisal in hospital nurses. Interviews were tape-recorded, transcribed and eventually listened by the primary investigator to assure accuracy of the transcription. Additionally, all three investigators listen again to the interviews. Through this process they gained enough preliminary insight to move onto reflecting on participants’ experience.

Each researcher highlighted the quota that captured each participant’s (a) feelings and (b) perceptions about (i) professional role, and (ii) personal, non-professional, entity. Notes were taken in relation to these four categories of data. Similar perceptions and feelings were then grouped. Subsequently, the three researchers came together and through ongoing reading, reflection and discussions, the common themes among them emerged and were grouped into: (a) participants’ feelings and perceptions about personal self-concept and worth appraisal, and (b) participants’ perceptions and feelings about professional self-concept and worth appraisal. These groups of initial categories of themes were further discussed and main themes and subthemes emerged. Researchers’ reflection and comprehension on the way the main themes and subthemes were associated revealed the core theme (van Manen, 1990; Boyd, 1993). Finally, the main themes and subthemes, as well as the core theme were brought back to the participants for confirmation. The above procedure and consensus among the three researchers regarding the final interpretation of the data, added to the rigor of the analysis.

Furthermore, all nine of Munhall (1994) criteria of research rigor were applied in the study. In particular, since the participants verified the interpretation of their narratives by the researchers in the third interview, this resulted in confirmation of the criteria of representativeness, reasonableness and recognizability. Moreover, prior to submission for publication, the participants received a copy of the study manuscript and approved its content regarding the quota included and their interpretation (confirmation of readability). Additionally, the participants assured during the third interview that they felt not only familiar with the data (recognizability criterion), but they also came across certain dimensions of the phenomenon that they had not reflected on before (raised consciousness and revelation criteria). Furthermore, during the presentation of the finding the participants discussed with the researchers the importance of the project and provided comments regarding implications for nursing (relevance and resonance criteria). Finally, the integrity of data analysis was partially assured by the credentials of all three researchers. In particular, the main researcher is an assistant professor in mental health nursing with long clinical experience as a nurse, academic experience in conducting phenomenological studies and a number of relevant publications. The second researcher is a psychologist and post-doctorate researcher with experience in phenomenological analysis and relevant academic publications. The third researcher is a psychologist with long experience in phenomenological analysis.

All interviews were carried out in Greek language. As a result, the findings and particularly the participants’ quota were translated and back-translated by two independent bilingual nursing scholars (EP, MK), as well as by a bilingual psychology scholar (AK), aiming to minimize meaning lost in the translation (van Nes et al., 2010). Disagreements were resolved by consensus, and were further validated by a professional translator.

## Results

### The Core Theme

Through the participants’ descriptions it was revealed that their personal self-perception could not be separated from the living experience of their professional role concept. In particular, as the participants began to talk about their professional role, they spontaneously began to describe themselves in their personal life and vice versa. Therefore, it appeared that the process of evaluating professional worth was directly and bidirectionally linked to personal worth assessment, while both of these processes seemed to be influenced by positive and negative feelings about their professional role concept, and vice versa.

In particular, participants’ narratives regarding feelings and perceptions of their self and their professional role, revealed certain important topics. The most prominent finding, identified as the core theme herein, was the interplay among the elements of professional worth appraisal, personal worth assessment and self-perception, constantly influencing each other.

”[…] giving through my job is quite strong and it gives me a feeling of job satisfaction […]. In that case I really feel that I am worthy as a human being in general.”

Established positive or negative self-perception seemed to influence professional role perception and professional worth appraisal. In particular, positive feelings about the self and personal competencies seemed to enhance the perception of effectiveness in clinical settings and adequacy of professional skills, resulting in increased professional worth perception.

“[…] I am very satisfied with myself, I am very satisfied with my work and my professional skills, too […]. I know my worth and through my work I can offer significant quality and quantity service.”

Nevertheless, participants described a two-sided interaction between the professional and the personal aspect of the self. Thus, experiences related to participants’ professional role were shown to affect the general idea participants had for themselves, including perception of personal worth.

“[…] regarding the way I feel about myself, this is what makes me feel stronger, more confident: when I look at a problem from all angles, and I struggle to solve it, and we make it (as a therapeutic team) in the end, and the positive outcome is evident. This makes my day, my confidence. Sometimes, of course, it all happens the other way around […].”

Moreover, while participants talked about themselves as individuals, two distinct ways of personal worth appraisal were prominent in their narratives. In particular, personal worth was based either on internal or external criteria, which further appeared to determine the appraisal process not only of their personal worth but also of their professional value. The issue of internal and external criteria is further described below.

#### A. Perception of the Professional Self-Concept and Professional Role-Based Worth Appraisal Process

I. Caring as the moral duty of offering one’s services

The central experience of the professional role of participants was their contribution to patient care. Their narratives revealed, as the central meaning of the living experience of their professional worth, their perception of nursing as a caring vocation, based on the moral duty of offering one’s services to the community. In more detail, participants perceived their professional role as offering support and quality care, cure and relief to patients. The moral duty of offering one’s services, as an integral part of the participants’ perception of their professional self-concept, was expressed in four different ways: (a) as a need for self-expression, (b) as an expression of social sensitivity, (c) as a means of empathy, and (d) as a means of professional satisfaction. **Box 1** depicts representative excerpts for each category.

**Box 1. The different aspects of participants’ perceptions of their professional self-concept and representative excerpts for each of them.**

***The moral duty of offering one’s services as a need for self-expression***

“[…] I really feel good, because I think that through my profession I can offer things; […] I think that one’s character has many aspects and that this profession fits my giving side; I can offer things to other people [the patients], I can reassure them, calm them down, be with them, make them feel better. It suits me well and I enjoy doing it.”

***The moral duty of offering one’s services as an expression of social sensitivity***

“[…] when it comes to my own family, if anything happens, I can offer them something, I don’t feel worthless […]. When I go out with friends […] and they have some small issues to address, I like to help them out in any way I can[…]. Everybody, in this life, can play their part, in any way they can.”

***The moral duty of offering one’s services as a means of expressing empathy***

“[…] I help people in need and it is nice to offer, to help someone […]. I witness the sickness and the suffering […]; I don’t like to feel pain, I can’t stand physical pain […] and I am trying to soften their pain […]. I feel content, when I can do something to ease their pain.”

***The moral duty of offering one’s services as a means of professional satisfaction***

“The sense of giving is quite strong and it gives me a sense of job satisfaction, but only from the moral side […].”

II. Appraisal of professional role worth and its link with positive feelings about one’s work

Furthermore, participants described internal and external criteria systems by which they evaluate their professional role worth. Participants who predominantly activate an internal criteria system (ICS) of evaluation, reported that receiving acknowledgment from a patient was a form of external assessment of their contribution to clinical outcomes. However, although this was considered as positive feedback, it was not necessarily a prerequisite for the living experience of positive feelings about their work, and subsequently for a high professional worth perception. In contrast, these feelings seemed to be associated more with the clinical outcome as evaluated by the participant him/herself.

”I do get satisfaction from my patients […]. Since most of them are not able to talk, they cannot express their gratitude directly […], so you do not always get the credit from them […]. You cannot always expect others to tell you ’thank you’ for what you do, but sometimes you do want to hear it […]. I get a moral kind of satisfaction when I see… in time, that the care I provided had a positive outcome, and that I have played a part in improving a patient’s health; that’s just so nice […].”

In contrast, in the case of a predominantly external system of criteria, receiving a verbal reward from patients was perceived as the main source of feedback, thus an important factor for the experience of positive feelings about work, and consequently of positive personal and professional worth perception.

”[…] when a patient asks me a question to which I happen not to know the answer, while one might think that that’s unacceptable on my part […], the whole thing immediately gets to me. It lowers my self-esteem […]. I get satisfaction only in relation to the reaction I get from patients […]. I get satisfaction from offering my services to patients in the hospital […]; when they smile […], that certainly raises my self-esteem and my job satisfaction is as high as it can get […]. It makes me sad when I have given my best to a patient and what I get back is an ungrateful reaction […].”

With regard to the main factors that affect the participants’ personal and professional worth perception, a treatment error during nursing care was the most important among them. This is highlighted in the following quote:

“[…] sometimes when your judgment is wrong, then there is a conflict within you and you keep asking yourself ’why wasn’t my judgment correct? Why didn’t I see it?”

“[…] when something bad happens at work […]; I think of it at home, whether it was me having done it all wrong.”

According to the descriptions of participants, the effect of treatment errors appeared to be determined both by the severity of the error and the nature of the criteria system. For people predominantly guided by an ICS, such circumstances worked as a motivation to face and overcome their limitations, given the error was not fatal. In contrast, for participants mostly dominated by an external criteria system (ECS), workplace errors, even non-fatal, and their conflict more often seemed to lead to feelings of guilt and distress than work correctively.

#### B. Personal Role-Based Worth Appraisal Process and the Perception of Personal Self-Concept

Similarly to the process of professional role-based worth appraisal, participants described in analogous way the procedure through which they evaluated their personal worth. In particular, through the participants’ narratives the living experience of personal worth appraisal could not be separated from their professional role, since as they began to talk about their professional role, they naturally began to describe their personal worth, and vice versa. Overall, it seemed that the system of internal and external criteria was a general criteria system the participants adopted during the development of their entity and the construction of their external image and formation of their behavior.

I. Internal and external criteria system for personal worth appraisal

Participants described two distinct pathways which seemed to lead to the appraisal of their personal role-based worth. Some participants mostly used internal, resilient criteria while others leaned on external, vulnerable ones.

*The ICS* seemed to entail norms attributed to oneself rather than the influence of the external environment. Participants who were generally characterized by an ICS seemed to mainly function autonomously and in a consistent manner. In that case, participants seemed to critically process external influences, i.e., opinions of others or stereotypes, without however, jeopardizing the image and personal worth perception they had of themselves, which seemed to be consistently positive and structured.

“[…] since I feel good about myself and I have this value system and I can recognize external stimuli, I accept them exactly as what they are and I activate an internal mechanism which filters them […].”

Participants also described an association between their feelings about themselves and the strong ICS they had established to appraise themselves, as drawn from the above quota, i.e., positive feelings and ICS. Furthermore, a participant with an ICS mentioned, while describing the source of his/her positive feelings:

“[…] When I undertake to do something and I complete it, that pleases me. End of story. In other words, it starts and ends with me.”

Overall, the catalytic role of an ICS and constructive self-awareness process for a positive personal worth appraisal is illustrated in the following excerpt:

“You will never manage to be highly appreciated by all, this is not possible; you will never succeed this because nobody is perfect and what I do and seems right for me, may be wrong for somebody else […]. Self-esteem is something you develop and is influenced by being aware of who you are and of your own potential as a person […]; when you realize your weaknesses and your strengths, your self-esteem will certainly rise. Because you are at peace with yourself and can admit to somebody else “yes, you do it better than me.” […] Although I am doing my best, sometimes I know I can do better. You never stop trying to improve, but it has no effect on the image you have of yourself.”

The opposite seemed to occur in participants mainly characterized by an ‘external’ system of criteria for appraising their worth. They described their self, mainly shaped by externally defined standards. Participants felt ‘obliged’ to meet those standards, in order to be accepted by society as a whole, as portrayed below:

“[…] The way I evaluate myself is associated with how people who are important to me appreciate me. Yes, I think I have difficulty in appraising myself independently […;] that comes out subconsciously […]; if I think about it afterward and it upsets me, I say “But why should they underestimate me so much, why can’t I figure out my worth on my own?” […] I believe that if I realized what I’ m capable of, I would feel much better about myself and have a much higher self-esteem.”

Thus, the *external criteria system (ECS)*, was mainly formed by external judgments, beliefs and attitudes, which were, however, able to affect participants’ perceptions and feelings, but not necessarily their personal qualities. Overall, participants employing mainly an ECS, did not appear to have formed a positive and stable self-perception. Rather, they seemed unable to filter social influences effectively, including those from significant others. As a result, they were unable to reject or appropriately incorporate outside information; nor could they build an independent system of benchmarks to filter social influences and help maintain a positive, relatively stable, self-perception.

The following excerpt depicts a participant’s perception of low self-worth and negative feelings about the self, attributed to an ECS:

“[…] when I look back at myself and my relationships with others, I put myself down. That is, I just can’t see how worthy I am, because I am interested in what other people think of me […]. I’m so easily affected by negative comments […]. I wish I didn’t place so much importance on the opinions of others and what I am to others […].”

Typically, participants attributing to themselves an external system criteria described that their attitude reflected values directed primarily at them from the outside, rather than through an endogenous process and belief system. As a result, they were experiencing their worth mainly through the comments coming from their environment.

II. The congruence between the self and the external image of the self and expressed behavior

During the course of the synergistic process of balance between the self and the external image of the self and subsequent behavior, if the participants failed to filter external influences through internal criteria, then the evaluation of their entity remained low and unstable.

“I allow myself to be influenced by others too much […]; (the opposite would be) to do something about it and not say afterward “Damn! Why didn’t I say what I thought was right? I should have said that, or this, etc.” […] At the end of the day, I am not very pleased with myself.”

Furthermore, participants with an external system of criteria, who seemed to experience a conflict between their self and the external image of their self and subsequent behavior, would become even more vulnerable to self-criticism and devaluation of personal self-worth. The above extract depicts the struggle between the self and the external image of the self and subsequent behavior, and its painful impact on the participant’s worth appraisal.

In contrast, participants with a powerful internal self-assessment criteria system did not appear to internalize influences from people or events they had not previously rated as significant. They would successfully balance the internal, stable system of self-assessment criteria with the processing of external stimuli, whether significant or not. All of the above are reflected in the excerpt below:

“[…] of course I value myself highly, both as a person and as a professional, and in terms of education and training. […] It makes no difference to me if I receive recognition from a person I don’t value as important. It matters only when the individual is special to me. […] My principles stem purely from my moral values. I still can’t tell if I inherited them from my parents or I have developed some during the course of my life, but they are very important to me […].”

“[…] when an incident that upsets me happens, I then try to think and assess myself, according to my standards […]. If I feel that I was honest, I say “I am at peace with myself,” meaning that what I believe about myself was consistent with my behavior.”

III. Continuous self-reflection as a means to personality development

From the participants’ descriptions, it appeared that, regardless of whether the evaluation mechanism was based on internal or external criteria, it was built on a continuous process of introspection and self-awareness including encounters and accomplishments. Although the establishment of an internal or ECS seemed to originate back in childhood, the system itself continued to evolve throughout the course of one’s life. In the following quota, participants describe how they perceive the gradual development of an internal and stable evaluation mechanism of self-worth and its effect on their life.

“[…] I feel that on a personal level I am gradually getting better, at least I try; I cannot say I’m completely satisfied, but I have reached a certain degree of self-awareness and I try to work on it, and, hopefully, to gradually improve as a person […]. I would say that my self-awareness has grown bigger over the years […].”

“[…] I am just slowly trying to complete whatever I have started, and I keep telling myself: “you’re okay, you’ve done well until now, so you can do more.” […] I want to be more confident, to think highly of myself, not to pay too much attention to what others think or how others see me. I think I’ve made considerable progress […]; years ago I would be much more vulnerable; low self-confidence, low self-esteem […]”

#### C. Personal and Professional Role-Based Perception of Worth: A Continuous Interaction

It was revealed through participants’ narratives that professional career advancements seemed to constitute a source of positive feelings not only for their professional role and professional worth appraisal, but also for the self, resulting in enhanced personal worth perception. This interaction seemed to be generally based on an ICS of personal and professional worth valuation.

“[…] regarding my personal worth, I feel really good about myself and this goes not only for now, it has been like that for the past 10 years, which were important years, because they were the start of my professional career; you start from nothing (at work) and gradually you build up your professional standing. I think I feel very good about that. I feel satisfied with how far I have come. Professional worth makes you feel different even in your personal life.”

Nevertheless, a perception of professional competence, reflecting a positive professional worth appraisal, was described as a prerequisite for enhanced personal self-value and feelings of satisfaction from the self.

“[…] I feel much better when something goes well at work. I get satisfaction from my work […]; the more capable I feel and the better I feel at work, the more satisfied I feel about myself and this interaction is evident in all phases of my life.”

Moreover, confirmation of personal worth through professional competence was described as a means of strengthening personal confidence as well as increasing the ability to cope with difficult work-related situations.

“My job gives me positive feedback many times. I know that I do things right, and I see that this has a positive impact on me.”

The following words of an ICU nurse illustrate the direct association between participants’ perception of personal worth and their clinical nursing role.

“I feel that I am a person of value when I see my patient doing well or when, for example, I diagnose a problem early. […] Your self-esteem comes from the patient and you see it in what you do and the impact of what you do.”

“[…] I am very satisfied with myself, I am very satisfied with my work and my professional skills, too […]. I know my worth and through my work I can offer significant services both quality- and quantity-wise.”

Participants also addressed the importance of a positive personal image and personal worth appraisal not only for an optimal professional standing, but also for positive professional role feelings.

“[…] I feel good about my work […]; to do this job you have to feel competent enough and fulfilled as a person, to be at peace with yourself, to have an inner balance […].”

Similarly, a perception of decreased self-worth was described as a factor which seemed to hinder one’s ability to appraise professional worth positively, leading to feelings of incompetency and to lack of self-acceptance regarding one’s professional image.

“I have a low self-esteem; I don’t think highly of myself at times […]. I often feel insufficient and cannot honestly picture myself as a good nurse […].”

The interaction between: (1) optimal professional worth appraisal and a positive professional role image on one hand, and (2) an ICS and a positive personal worth appraisal on the other, is highlighted in the following quote:

“I am able to say that I enjoy a good status as a critical care nurse, I feel that I can effectively manage multiple problems and different kinds of emergencies in my job […]. I build my self-esteem from the way I feel, how satisfied I am with myself and the feedback I get from people I think highly of.”

Overall, the above quote stresses the importance of personal feelings about one’s “outer” self as well as the input from significant others in creating a positive personal worth perception.

Most importantly, the significance of a stable, positive self-worth perception as a buffer system against disappointments and devaluation in the workplace, along with an ICS, is evident in the following quota:

“I don’t think there is anything strong enough to disrupt the relationship I have with myself […]. I am not the kind of person who will become devastated if something bad happens at work […]. Okay, some of them (the patients) do behave as if you are totally worthless. But I know that what I do, I do it because I want to, and, no matter what they say, I am who I am and I will not change.”

“[…] they may laugh at you, they may try to put you down, but I’ve been through all that before. I think it reflects their personal problems, or whatever […]; it is not my job to deal with it […]. I know who I am, I have my own beliefs, I will not let anyone try to change the way I am and destroy me[…]; what counts most for me is not to let anybody mess with my personality. I am at peace with myself. I have my own values and I will stand up for them.”

## Discussion

The aim of the present study was to explore the living experience of critical and emergency nurses regarding their professional role concept and how this was associated with perceptions and feelings about personal and professional role-based worth. The findings revealed that personal and professional self-concepts were interlinked and constantly influencing each other, as were personal and professional worth appraisal. Moreover, the participants’ narratives illuminated the existence of two distinct self-assessment criteria systems, i.e., an internal and an external one, which were likely to be activated at a personal and professional level when challenged with conflicting conditions. Participants with an internal criteria evaluation system displayed a steady image of themselves and their professional role, while participants with an ECS seemed vulnerable to external adverse events and negative opinions expressed by others.

One example of the difference between the two systems was in relation to the living experience of offering one’s services through the profession, which was a central dimension in professional self-concept. For participants predominantly influenced by an ICS, offering their nursing services was a need for self-expression as well as an embedded characteristic in themselves. Most importantly, offering one’s services was assessed by the participants themselves according to the clinical outcome of the patients, which was the primary source of job satisfaction, also serving as a buffer system against adverse work-related phenomena. On the other hand, for participants displaying ECS assessment, positive reinforcement from their environment and mainly from patients, through direct verbal and non-verbal expressions, was the crucial element for positive feelings and worth perception at a personal and professional level.

This finding may support a set of implications regarding the clinical placement of nurses (Emeghebo, 2012): nurses mainly guided by an ECS may be well advised to follow clinical specialties where clinical outcomes are evident in a short time period and/or adverse phenomena are less frequent. For instance, obstetrics, child healthcare or serving at a nursing school, respectively, might be a suitable choice. In contrast, nurses mainly functioning under an ICS might be more suitable for emergency and critical care nursing settings, in which external feedback from patients or their families is less direct. As for nurses already working in specific clinical settings, especially designed interventional programs aiming to psychologically and organizationally empower vulnerability to job distress, and burnout are proposed. Such interventions could mostly focus on nurses who mainly display an ECS and are employed in clinical settings characterized by futile care and frequent experiences of frustration, such as the ICUs or the psychiatric wards (Austin, 2016).

Additionally, regarding the way a set of self-assessment criteria could affect the personal and professional self-concept in nurses, as reported herein, one may also underline the following: the self-assessment criteria system seems to be a personality trait, originating in one’s early experiences, which further affects the way people perceive and evaluate themselves and their relationships with others (Bornholt and Piccolo, 2005). Given that personality traits are quite resistant to change, nurses with an ICS will always be more confident and at peace with themselves compared to nurses with an external criteria assessment system (Abbott et al., 2008; Sim et al., 2014). However, the work environment of nurses constantly sets challenges for them which may influence their professional self-concept (Emeghebo, 2012). Thus, the organizational parameters of the work environment, apart from being relevant to the quality of care and patient safety outcomes, may be viewed as important factors associated with nurses’ overall well-being and personal self-concept. Additionally, this might inform interventions toward nurses with negative self-concept and thus susceptible to psychological distress, even anxiety and depressive symptoms, who have been placed in clinical settings characterized by challenges to one’s self-esteem, e.g., limited participation in decision making or by a high frequency of morally distressing situations (Oh and Gastmans, 2015). Nevertheless, extensive literature shows that inequalities in the work environment, such as low status in nurses compared to physicians, restricted initiative in decision making, moral challenges, long working hours, low salaries and a negative public image of nurses, can lead nurses to feel undervalued at work (Read and Laschinger, 2015). In addition, the aforementioned factors are related to higher nurses’ job stress, low job satisfaction and low job retention rates (Botha et al., 2015). Based on these data, one might argue that nurses with an ECS might be more likely to be affected at a personal and professional level by these factors, thus be more vulnerable to adverse phenomena regarding professional status and overall well-being and subsequently dysfunctional professional attitudes, e.g., work dissatisfaction or absenteeism and resignations (Hensel and Stoelting-Gettelfinger, 2011).

Moreover, predominance of an ECS may even magnify existing problems in the work environment resulting in negative professional self-concept and further distress. Judge et al. (2003) suggested that the core self-evaluation process affects the way an individual interprets and reacts to the organizational status of a given environment, as well as to relevant challenges. Therefore, a dysfunctional self-worth appraisal system may contribute to elevated job stress and burnout, implying the need for supportive interventions for these group of nurses. In particular, several studies have reported an association between self-concept and burnout. For example, Cao et al. (2015, 2016) reported that positive professional self-concept resulted in reduced emotional exhaustion and depersonalization besides increased professional accomplishments in Chinese hospital nurses (Cao et al., 2015, 2016). Best et al. (2005) found that employees with a perception of low core self-evaluation perceived their work settings as stressful and were at a higher risk for burnout manifestation than those with higher core self-evaluation perception. Spence Laschinger and Finegan (2008), in a longitudinal study with nurse managers experiencing emotional exhaustion, showed that the quality of core self-evaluation process was an important contributing factor associated with the burnout syndrome. These findings may suggest that burnout interventions need to not only address situational factors but also recognize the importance of employees’ belief systems and self-appraisal schemes in interpreting work conditions. At the same time, these findings suggest that a supportive work environment may have a positive impact in nurses’ positive work attitudes through boosting their personal and professional esteem.

Indeed, studies on organizational commitment provide data on how nurses’ professional self-concept may be associated with their resilience to stress and their overall well-being. Organizational commitment is a construct strongly related to professional role perception (Cao et al., 2015), entailing one’s psychological attachment to an organization and desire to remain in the organization (Meyer and Allen, 1991; Cao et al., 2015). The study by Cao et al. (2015) revealed that a positive professional self-concept was linked with enhanced organizational commitment and decreased symptoms of work-related stress and burnout. Organizational commitment was identified as a buffer against the association between negative professional self-concept and burnout intensity in this study (Cao et al., 2015). Based on this, enhancement of nurses’ organizational commitment through interventions targeted to develop a supportive and empowering work environment may boost a positive professional self-concept among them, and further advance their resilience to work-related stress and subsequently the quality and safety of provided care.

Apart from the above, it is important to mention the connection between a positive or negative professional self-concept and clinicians’ efforts to preserve quality and safety of care. In a study by Andrews et al. (2011), the narratives of 106 staff nurses revealed that those participants who were experiencing contradictions and unmet expectations related to their professional role described feelings of powerlessness, isolation, and low self-esteem. These feelings reflecting diminished professional image seemed to affect the participants’ perceived ability to provide quality patient care and ensure patient safety. And vice versa, perceived inability to act in a professionally autonomous manner on the patients’ behalf influenced negatively the participants’ professional self-concept. Moreover, since nurses are strongly inclined to report patient safety problems play an important role in ensuring patient safety (Carayon et al., 2006). Based on this, one may argue that nurses with a positive personal and professional concept or mainly characterized by ICS may be more willing to advocate for patients’ rights and report patient safety issues. Thus, supporting nurses to develop a positive professional self-concept may have an effective impact on patient safety issues.

In line with the above are data coming from studies on nurses’ professional engagement, a crucial dimension of nurses’ professional self-concept which has been related to patient safety (Teng et al., 2009). Professional engagement reflects one’s assignation to the ethical code of the profession and commitment (a) to professional values and goals, (b) to contribute significantly to the profession, and (c) to remain part of it (Lu et al., 2007; Teng et al., 2009). Teng et al. (2009) found that nurses’ professional engagement was positively associated with enhanced patient safety in terms of patient falls, medication errors, incomplete or incorrect documentations and delayed care. In addition, nurses’ professional engagement was also positively associated with better patient-perceived quality of care, regarding responsiveness and empathy. Thus, interventions aiming to preserve a positive professional self-concept may increase professional engagement and through that may have a positive impact on quality and safety patient care standards.

With regard to clinical implications toward a supportive and empowering work environment, Best et al. (2005) recommended interventions that promote person-job fit such as necessary resources to facilitate goal achievements, as well as ergonomic work environment to ensure that efforts are rewarded and autonomy and control over practice are achieved. In addition, novice strategies in caring, communication and leadership skills training may be effective interventions toward this goal. It has been reported that, self-awareness education and communication training techniques regarding assertive and self-confident behavior may enhance nurses’ professional self-concept (Unal, 2012). Given that caring through offering one’s services is in the core of nurses’ professional self-concept it is necessary to support education in advanced clinical roles, along with continuous educational activities so they are able to keep their positive professional self-concept and a perception of quality patient care.

### Limitations

The main limitation of the present study regards the fact that data collection interviews were carried out in Greek language, which may have resulted to some degree to meaning lost. However, all suggestions provided by van Nes et al. (2010) were followed herein to assure minimum bias. Furthermore, although this study took place in the metropolitan area of Athens, it should be noted that the majority of Greek hospitals have similar organizational and healthcare policies. Thus, the present results may be adequately generalizable to the rest of Greece. Nevertheless, caution is needed regarding the interpretation of these findings regarding other healthcare settings, or male nurses, since the great majority of the participants were females. Additionally, no inferences are possible about the rigor of responses related to the self-worth appraisal due to the subjective and complex nature of this phenomenon.

## Conclusion

This study highlighted that the way nurses feel and think about themselves is associated with the way they experience their professional role and vice versa, and that professional role-based self-concept and professional worth perception can be linked with their well-being. Additionally, the present study emphasized the way self-evaluation criteria system may be associated with the personal and professional self-concept in nurses. Thus, it is important for the hospital managers to provide nurses with all necessary resources to help them sustain optimal perceptions and feelings about themselves personally and professionally. Overall, interventions directed to change work conditions rather than dispositional and personality factors are considerably more realistic; however, the present results suggest that both hospital and nurse managers must also take nurses’ perceptions of self-concept into account when designing healthy and productive work environments in healthcare. Thus, interventions toward work-related stress and burnout alleviation need to not only address situational factors but also recognize the importance of employees’ belief systems and self-appraisal schemes in interpreting work conditions. Special focus is proposed on nurses with maladaptive personal or professional self-concept as susceptible to psychological distress. In that case, nurses’ placement in clinical settings characterized by work environment inequalities and challenges to one’s personal and professional esteem might be ineffective. Interventions that promote person-job fit seem to facilitate goal achievements, and to further ensure that efforts are rewarded and both autonomy and control over practice are achieved. Thus, a supportive and empowering work environment may promote a positive professional self-concept among healthcare professionals, and further improve their resilience to work-related stress and subsequently the quality and safety of provided care.

## Authorship Statement

All authors listed meet the authorship criteria according to the latest guidelines of the International Committee of Medical Journal Editors. Moreover, all authors are in agreement with the manuscript.

## Author Contributions

MK: study design, manuscript preparation, and data analysis. AK: data analysis, editing, and critical review. KD: data analysis. MG: critical review. EP: study design, data analysis, and critical review.

## Conflict of Interest Statement

The authors declare that the research was conducted in the absence of any commercial or financial relationships that could be construed as a potential conflict of interest.
